# Integrated ^18^FDG PET/CT: Utility and Applications in Clinical Oncology

**DOI:** 10.4137/cmo.s504

**Published:** 2008-09-19

**Authors:** Inmaculada Pinilla, Beatriz Rodríguez-Vigil, Nieves Gómez-León

**Affiliations:** Department of Radiology, Hospital Universitario La Paz, Madrid, Spain

**Keywords:** PET, PET/CT, FDG, oncology, cancer

## Abstract

Accurate diagnosis and staging are essential for an optimal management of cancer patients. Positron emision tomography with 2-deoxy-2-fluorine-18-fluoro-D-glucose (^18^FDG-PET) and, more recently, ^18^FDG-PET/computed tomography (^18^FDG-PET/CT) have emerged as powerful imaging tools in oncology, because of the valuable functional information they provide. The combined acquisition of PET and CT has synergistic advantages over its isolated constituents and minimizes their limitations. It decreases examination times by 25%–40%, leads to a higher patient throughput and unificates two imaging procedures in a single session. There is evidence that ^18^FDG-PET/CT is a more accurate test than either of its components for the evaluation of various tumors. It is a particularly valuable tool for detection of recurrence, especially in asymptomatic patients with rising tumor markers and those with negative or equivocal findings on conventional imaging tests. Yet, there are some limitations and areas of uncertainty, mainly regarding the lack of specificity of the ^18^FDG uptake and the variable ^18^FDG avidity of some cancers. This article reviews the advantages, limitations and main applications of ^18^FDG-PET/CT in oncology, with especial emphasis on lung cancer, colorectal cancer, lymphomas, melanoma and head and neck cancers.

## Introduction

Cancer is one of the leading causes of death worldwide. Accurate diagnosis, staging and restaging are essential for an adequate therapeutic management of these patients. Conventional imaging techniques, such as computed tomography (CT) and magnetic resonance rely on anatomic alterations and abnormal contrast enhancement, with limitations to detect infiltration in normal-sized structures and characterization of residual lesions. Positron emision tomography (PET) with 2-deoxy-2-fluorine-18-fluoro-D-glucose (^18^FDG), an analogue of glucose, provides valuable functional information based on the increased glucose uptake and glycolysis of cancer cells. Therefore, PET has the ability to depict metabolic abnormalities before morphologic alterations occur. The main drawback of PET is the limited spatial resolution which impedes precise localization of foci of ^18^FDG uptake and hinders identification of lesions adjacent to organs with physiological ^18^FDG uptake (urinary tract, bowel) (Shreve et al. 1999). In addition, ^18^FDG is physiologically taken up by various organs and also by sites of inflammation. The hybrid PET/CT modality acquires PET and CT data in the same imaging session and allows accurate anatomic localization of the lesions detected on the ^18^FDG-PET scan. Since its introduction integrated PET/CT has rapidly gained clinical acceptance and, in the last decade it has become an important imaging tool in clinical oncology.

In this article, we review the advantages, limitations, clinical utility, and main applications of combined ^18^FDG-PET/CT in oncology.

## General Aspects of Combined PET/CT

### Advantages of combined ^18^FDG-PET/CT

There are several advantages of combined PET/CT over its isolated constituents, that translate into highly synergistic benefits in the management of a variety of cancers.

#### Technical advantages

From a technical point of view, the use of CT data photon attenuation correction instead of data from an external radioactive transmission source results in a much faster PET image acquisition, decreasing examination times by 25%–40% relative to stand-alone PET (Hany et al. 2002; von Schulthess et al. 2006). Straightforward consequences are a higher patient throughput and a more efficient use of the fast-decaying PET radiopharmaceutical (Hany et al. 2002). In addition, owing to the high photon flux used for CT attenuation correction the noise is reduced and the image quality of the PET scan is improved (Carney et al. 2003).

#### Clinical advantages

For the patient, the decreased scan time and unification of the two imaging procedures in a single session, performing preparation such as fasting only once, lead to a greater patient comfort and convenience.

**Figure 1:**
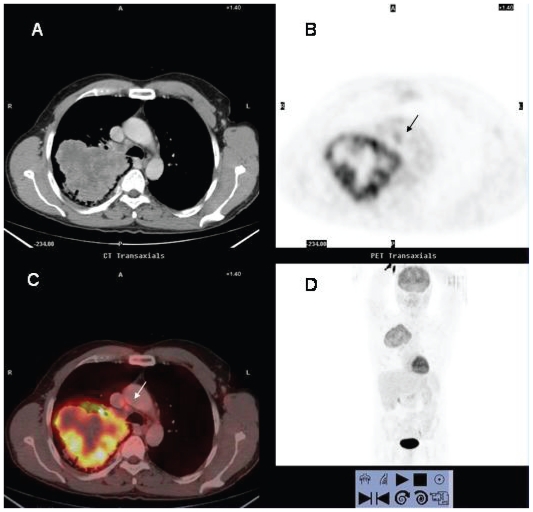
^18^FDG PET/CT study of a 60 year-old man with NSCLC. There is a large mass in right upper lobe that exhibits peripheral hyper-metabolic activity consistent with tumor viability and central photopenia suggesting necrosis of the tumor. A small lymph node in the right lower paratracheal area with increased ^18^FDG uptake is observed (arrow). Histological examination demonstrated metastatic infiltration.

The most remarkable clinical advantage of the integrated PET/CT is the accurate co-registration of metabolic and anatomic data, providing improved lesion localization and characterization, which results in a significant reduction of false-positive and false-negative findings, and increased diagnostic and staging accuracy of numerous cancers as compared with either modality (Charron et al. 2000; Hany et al. 2002; Israel et al. 2002; Antoch et al. 2003; Bar-Shalom et al. 2003, Bristow et al. 2003; Czernik et al. 2003). In general, the CT component adds mainly specificity, whereas PET adds mostly sensitivity. Thus, the combined PET/CT is a more sensitive and specific test than either of its constituents obtained separately (von Schulthess et al. 2006). Other benefits of PET/CT include identification of small lesions that might be overlooked on either PET or CT alone, normal-sized malignant nodes (Fig. 1), confident characterization of suspicious or equivocal findings on other imaging techniques, and biopsy guidance. In addition, the gathered functional and anatomic data are integrated by the interpreting physicians in a single report, with a diagnostic impression formulated from the combination of both techniques, that facilitates the transfer of diagnostic information to the referring physicians. It must be noted that the quality of the CT component is variable depending on the institution and clinical indication for PET/CT. Hence, the PET/CT report should specify whether the CT scan was performed with a very low current (such as 10 mAs) or low current (40–80 mAs) for attenuation correction and anatomic localization only, or with full dose for diagnostic purposes, and the use of contrast agents, to decide the need for further morphologic imaging. In addition, because of the synergistic benefits of the combined acquisition of both modalities, the administration of intravenous contrast material can be eliminated for certain indications (e.g. monitoring response to therapy) (Wong et al. 2007). These benefitial aspects of PET/CT potentially improve the management of cancer patients.

### Limitations and pitfalls of combined ^18^FDG-PET/CT

There are potential pitfalls of PET/CT related to technical factors. Patient motion during imaging acquisition may produce misregistration on the fused images and cause confusion or mistakes regarding the correct localization of the ^18^FDG uptake (Kapoor et al. 2004). Although patient movement and breathing motion can be minimized by placing the patient in a comfortable position and acquiring CT in normal expiration, some artifacts caused by respiratory, cardiac or bowel motion are unavoidable. These mismatchs are readily identified by carefully reviewing both sets of images, and usually do not create diagnostic dilemmas. A drawback of acquiring the CT in expiratoty phase instead of instead of full inspiration is that the chest images show, compared with diagnostic thoracic CT scans, lower lung volumes and more dependent atelectasis and ground-glass opacities that potentially obscure small nodules. Even though, the chest images of PET/CT are usually of an adequate quality for most of the oncologic indications (Wong et al. 2007). In selected cases an additional inspiratory thoracic CT can be performed after the acquisition of PET/CT in expiration. Attenuation correction artifacts that may occur with high-density elements such as metallic devices are easily recognized on uncorrected PET images, which are always available.

**Figure 2A y 2B:**
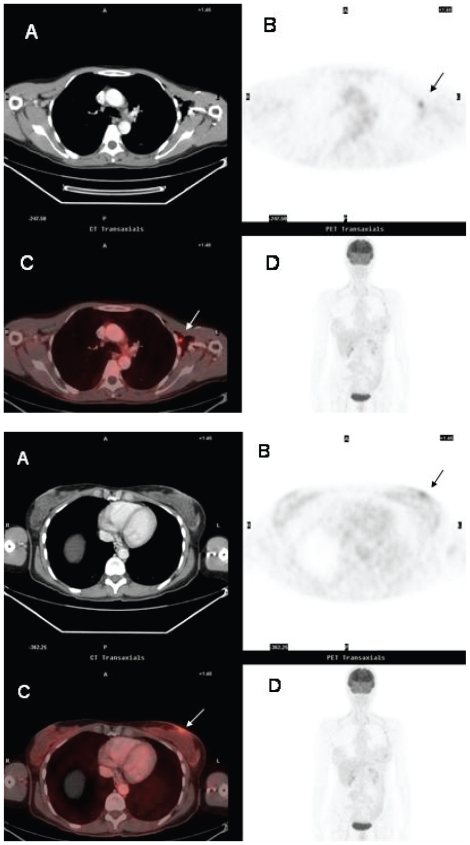
False-positive FDG-uptake in viral infection. Forty-seven year-old woman referred for staging of T-cell non-Hodgkin’s lymphoma. ^18^FDG PET (**B, D**) ^18^FDG PET/CT fusion (**C**) scans shows abnormal foci of ^18^FDG uptake in the left axilla (**2A**) (arrow) and the skin overlying the left breast (**2B**) (arrow). The findings were misinterpreted as cutaneous and nodal lymphomatous infiltration. Skin and axillary node biopsies demonstrated herpes virus infection and benign reactive follicle hyperplasia respectively. There was no evidence for malignancy.

Limitations of ^18^FDG-PET/CT in the evaluation of cancer have been documented as well. ^18^FDG is not a cancer-specific tracer and accumulates in areas of increased metabolism such as several normal organs (brain, salivary glands, vocal cords, myocardium, urinary tract), and brown fat. Although the better anatomic localization and morphologic information of ^18^FDG-PET/CT improves the diagnostic accuracy compared to PET standalone, in occasions, tumor detection may be impaired in these structures, even on ^18^FDG-PET/CT. In this regard, relatively small brain metastases can be missed on ^18^FDG-PET/CT owing to the high background activity. Hence, symptomatic patients with negative scans or those at high risk for brain metastases will require further imaging with MRI or contrast-enhanced CT. ^18^FDG is also taken up by activated leukocytes and macrophages, resulting in enhanced uptake in sites of active inflammation and tissue repair (infection, sarcoidosis, vasculitis, post-radiotherapy and post-surgery changes, etc) (Fig. 2). The findings on the CT component may facilitate the interpretation of ^18^FDG-avid lesions, e.g. the morphologic changes secondary to a bone fracture. However, exclusion of malignancy may be impossible based on ^18^FDG-PET/CT images only, and correlation with clinical data is essential to the correct interpretation of scans. In addition, a variety of benign tumors in the head and neck and colonic adenomas may exhibit an increased uptake.

**Figure 3:**
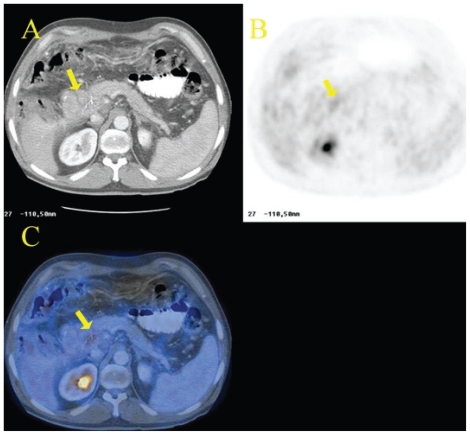
Recurrence of cholangiocarcinoma in a patient who had undergone surgical tumor resection. The CT scan (**A**) shows a mass at the site of previous surgery in contact with the pancreatic head (arrow). The lesions shows minimal ^18^FDG uptake on ^18^FDG PET (**B, D**) and ^18^FDG PET/CT fusion (**C**) images (arrow).

On the other hand, there are limitations related to the variability in ^18^FDG uptake of several types of cancers. Well-differentiated, hypo-cellular and mucin-producing tumors (and their metastases), such as bronchioalveolar carcinoma, hepatocellular carcinoma and intraductal papillary mucinous tumor exhibit low ^18^FDG uptake (Berger et al. 2000) (Fig. 3). Yet, this limitation of ^18^FDG-PET/CT may be valuable in certain cases, as the degree of ^18^FDG uptake may be correlated with the biological aggresiveness with a prognostic significance, and help to select therapies (Yang et al. 2005).

### Clinical utility

^18^FDG PET/CT has proven effective for diagnosis and staging of various cancers (Beyer et al. 2000), especially non-small-cell lung carcinoma (NSCLC), lymphoma, recurrent colorectal carcinoma, melanoma and sarcomas. Recently, especial attention has been focused on restaging and monitoring tumor response, where this technique is probably most useful (Oriuchi et al. 2006). ^18^FDG PET/CT is a valuable tool for detection of recurrence, particularly in asymptomatic patients with rising levels of tumor markers and also in patients with negative or equivocal findings on conventional imaging tests. In patients with residual masses after therapy completion or with post-surgical or post-radiation anatomic distortion, ^18^FDG PET/CT allows accurate differentiation between viable tumor and necrosis or scar. With its precise anatomic correlation, it may be used to direct diagnostic biopsy to the specific site of ^18^FDG uptake to obtain tissue confirmation of recurrence. Thus, relapses can be early detected and treated, while tumoral burden is still low. Conversely, exclusion of recurrence in areas of post-therapy morphologic abnormalities avoids unnecessary diagnostic procedures and treatments. However, studies performed within 2–3 months of radiation or 1–2 months of surgery may yield false-positive findings, as post-therapeutic inflammation causes ^18^FDG uptake (Hojgaard et al. 2007; Oriuchi et al. 2006; Von Schulthess et al. 2006).

Because metabolic changes in a tumor precede size reduction, ^18^FDG PET/CT is effective for assessment of response to therapy. Successful chemotherapy decreases cellular glucose transport and glycolysis and, hence, tumor uptake of ^18^FDG. It has been reported that a decrease in ^18^FDG uptake may be observed as early as 1–2 weeks after the first cycle of effective chemotherapy (Brun et al. 2002; Kostakoglu et al. 2002; Weber et al. 2003). In this way, ^18^FDG PET/CT enables early identification of non-responders and a change in therapy.

Available information in the literature on the impact of ^18^FDG PET/CT on radiotherapy treatment planning is limited (Van Baardwijk et al. 2006; Greco et al. 2007). However, current data suggest an improvement in target volume delineation. In this way, ^18^FDG PET/CT may modify radiotherapy fields to reduce radiation dose to normal tissues and allow selective dose escalation to hypermetabolic areas within the tumoral mass. The advantages of ^18^FDG PET/CT seem to be more relevant in lung cancer, enabling differentiation between tumor and atelectasis and detection of unsuspected metastatic lymph nodes, and in head and neck carcinomas, where a better delineation of involved sites may reduce sequelae of radiation. Further research is needed to determine the exact role of ^18^FDG PET/CT in radiotherapy treatment planning, however.

### Radiation dose

There is some concern regarding the higher radiation exposure of ^18^FDG PET/CT in comparison to PET standalone, especially in oncologic patients who will undergo repetitive scans for tumor follow-up. Tipically, a PET scan with 370 MBq (10mCi) of ^18^FDG delivers a dose of approximately 11 mSv to a patient, predominantly owing to the positrons emitted from isotopes. This dose is comparable to that of a diagnostic CT, ranging from 10 to 20 mSv. With the use of a CT scan for attenuation correction and anatomic coregistration the patient receives an additional dose that will vary depending on the quality of the CT scan and the protocol used: low-dose unenhanced-CT (LD-CT), full-dose contrast-enhanced-CT (FD-CT). A LD-CT with 40 mAs adds approximately 2 to 8 mSv, resulting in a final dose of 13–20 mSv for an integrated LD-^18^FDG PET/CT study, which is similar to a diagnostic contrast-enhanced CT. A LD-^18^FDG PET/CT performed with 40–80 mAs yields an adequate image quality and may suffice for many, though not for all, ^18^FDG PET/CT oncologic applications (see below) (Brix et al. 2005; Kneifel et al. 2003). Nevertheless, the risk-benefit ratio has to be taken into account in the individual patient, as correct diagnosis, staging and restaging are essential for an optimised and individualised therapy.

## Major Indications of ^18^FDG PET/CT in Oncology

### Solitary pulmonary nodule (SPN)

**Figure 4:**
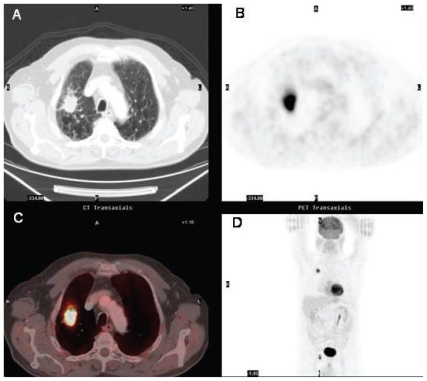
70 year-old man referred for evaluation of a pulmonary solitary nodule evaluation. Axial CT scan shows a 2.8 cm pulmonary lung in the right upper lobe with spiculated margins and emphysema. ^18^FDG PET and ^18^FDG PET/CT fusion images reveal increased ^18^FDG uptake in the nodule. The lesion corresponded to a squamous-cell carcinoma.

**Figure 5:**
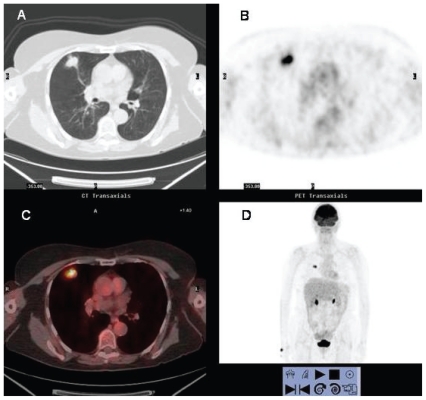
68 year-old woman that presented with a pulmonary node at chest radiograph. A nodule with pleura tail and increased ^18^FDG uptake is observed in the right upper lobe at ^18^FDG PET/CT scan. The histologic study demonstrated tuberculoma.

^18^FDG PET/CT helps characterize SPN, as most of malignant nodules show increased glucose metabolism (Fig. 4). The diagnostic accuracy of ^18^FDG PET depends on the size of the nodule and its avidity for ^18^FDG, and false-negative studies have been reported in nodules smaller than 1 cm, well-differentiated adenocarcinoma, bronchialveolar cell carcinoma, and carcinoid (Higashi et al. 1998; Gould et al. 2001). False-positive findings include infectious and inflammatory processes such as tuberculosis (Fig. 5), fungal infections, and sarcoidosis. It has been reported that ^18^FDG PET/CT can reliably characterize SPN ≥ 7 mm (sensitivity 97%, specificity 85%, overall accuracy 93%) (Kim et al. 2007), providing valuable information to guide patient management, especially useful where biopsy is risky, in elderly patients, or when there is a low risk for malignancy. This, SNPs with increased ^18^FDG uptake are likely malignant and should undergo further invasive resection or biopsy. However, nodules PET negative still need to be followed (usually by CT) because of the possibility of false-negative PET finding (Christensen et al. 2006).

### Non-small cell lung cancer (NSCLC)

^18^FDG PET/CT has an important role in the initial staging, restaging, and in radiotherapy planning. Advantages of combined ^18^FDG PET/CT over its isolated components in NSCLC evaluation include: better lesion identification and localization, higher detection rate of lesions with low ^18^FDG affinity, and depiction of tumoral infiltration in small lymph nodes.

**Figure 6:**
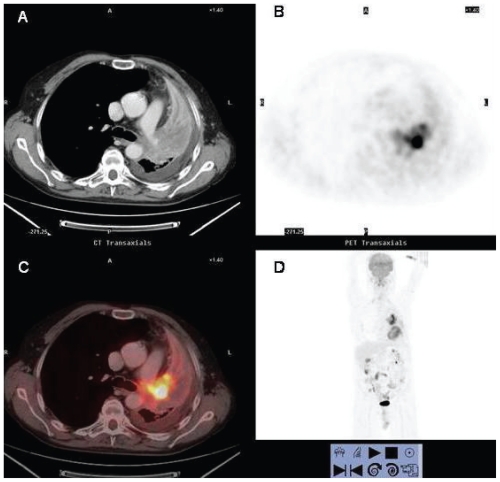
58 year-old man who underwent contrast-enhanced integrated ^18^FDG PET/CT for staging of newly diagnosed squamous carcinoma. Axial contrast-enhanced CT (**A**), axial (**B**) and coronal (**D**) PET and fused enhanced ^18^FDG PET/CT (**C**) images. Integrated enhanced ^18^FDG PET/CT (**C**) depicts the left upper lobe central ^18^FDG avid mass within the non-hypermetabolic collapsed lung parenchyma and allows differentiation between tumor and surrounding post-obstructive atelectasis. Thanks to the contrast-enhanced CT component of ^18^FDG PET/CT it is possible to delineate infiltration of the mediastinum and left pulmonary artery, classifying the tumor as a T4 stage. A left pleural effusion without ^18^FDG uptake is also noted. Accurate delineation of the tumor is important for radiation therapy planning.

**Figure 7:**
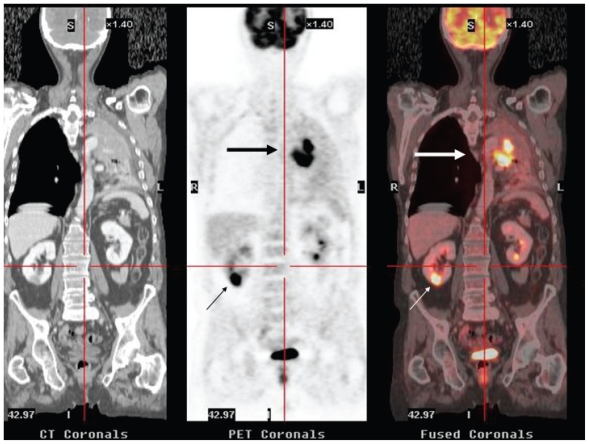
58-year-old man who underwent ^18^FDG PET/CT for initial staging of NSCLC. Coronal CT, PET and fused ^18^FDG PET/CT images depicted a intraparenchymatous right renal lesion with intense ^18^FDG uptake which were histologically proved to be metastatic disease (small arrows). The central upper left lobe pulmonary mass with lung atelectasis is also observed (large arrows).

^18^FDG PET/CT has been reported to be the most accurate imaging technique in staging NSCLC, with accuracies for tumor (T), nodal (N) and metastases (M) staging of 70%–97%, 78%–93% and 83%–96% respectively (Lardinois et al. 2003; Sachelarie et al. 2005). The most important benefit of the integrated modality relates to T staging, where ^18^FDG PET/CT is clearly superior to either of its constituents, mainly due to the precise anatomic localization of the ^18^FDG uptake (Lardinois et al. 2003; Halpern et al. 2005; Shim et al. 2005). Thus, CT improves depiction of focal chest wall and mediastinum infiltration and vascular invasion (Lardinois et al. 2003), whereas PET is useful in differentiating tumor from post-obstructive atelectasis (Fig. 6) and characterizing pleural effusions as malignant (Schaffer et al. 2004; Lavrenov et al. 2005; Devaraj et al. 2007). It should be stressed the need to perform a diagnostic CT with intravenous iodinated contrast material in order to achieve a precise definition of tumor extension, distinguish contiguity of tumor and mediastinum from the direct invasion of the walls of mediastinal structures, and depict vascular invasion (Lardinois et al. 2003; von Schulthess et al. 2006; Pfannenberg et al. 2007). This is of utmost importance for both, planning of 3D conformal radiotherapy and extended non-conventional surgery. Recently, Pfannenberg et al. found that contrast-enhanced PET/CT more accurately assessed the TNM stage in 8% of patients with advanced NSCLC compared with non-contrast PET/CT, and showed significant additional findings in 20%. They suggest that contrast-enhanced PET/CT should be performed in all patients with NSCLC who are primarily considered for local therapy such as surgery, neoadjuvant radiochemotherapy or definitive radiotherapy (Pfannenberg et al. 2007). Although PET/CT also has a higher diagnostic accuracy than either CT or PET alone for N staging (Lardinois et al. 2003), the improvement with respect to PET alone is more modest. The benefit lies in a higher specificity of PET/CT attributed to the precise anatomic information provided by the CT component. This is particularly useful for localization of lymph node metastases in patients with a mediastinal shift of with small solitary nodes that could be difficult on PET alone (von Schulthess et al. 2006). ^18^FDG PET and ^18^FDG PET/CT have a high negative predictive value for nodal involvement, greater than 90% (Pieterman et al. 2000; Schrevens et al. 2004, Pozo-Rodríguez et al. 2005). However, false-negatives due to micrometastases (“minimal N2 disease”) can occur in up to 8% of patients, although these patients have a better prognosis (Schrevens et al. 2004). The value of ^18^FDG PET/CT in nodal staging is limited by the low positive predictive value caused by inflammatory changes in lymph nodes, especially in geographic areas with a high prevalence of granulomatous disease. For this reason, it is necessary to obtain histologic confirmation of positive lymph nodes that would preclude surgery (De Langen et al. 2006). ^18^FDG PET performs well at depicting extrathoracic metastases and detects unsuspected metastases in up to 28% of patients with NSCLC (Eschmann et al. 2002; Lardinois et al. 2003) (Fig. 7). The CT component provides exact localization of the PET findings as well as complementary morphologic information that is especially useful in cases of doubtful adrenal lesions. In this regard, measurement of the Hounsfield Units of the lesion on the non-enhanced CT images (which are usually available) with the possible addition of a delayed enhanced CT at the end of the exploration may help characterize doubtful lesions, such as adenoma depicting ^18^FDG uptake (Elaini et al. 2007; Metser et al. 2006; Pfannenberg et al. 2007).

On the other hand, it should be noted that ^18^FDG PET/CT is not a sensitive technique for the detection of brain metastases because of the difficulty in depicting ^18^FDG-avid lesions in the physiologically hypermetabolic brain parenchyma (Bruzzi et al. 2006; Devaraj et al. 2007). For this reason, further imaging with brain MRI may be needed.

^18^FDG PET/CT also has a significant value in the assessment of suspected relapse, being particularly useful in cases with postherapy anatomic distortion (Keidar et al. 2004).

### Colorectal cancer

**Figure 8:**
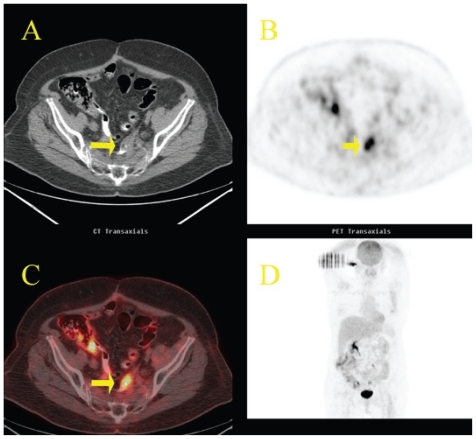
Local recurrence of colon cancer. Fifty-one year-old woman, status post-resection of sigmoid adenocarcinoma, referred for reevaluation. Axial CT (**A**) scan shows a nonspecific small soft-tissue mass at the site of previous surgery (arrow). Axial ^18^FDG PET scan (**B**) reveals focal intense accumulation of ^18^FDG in the pelvis that is difficult to localize anantomically. Axial fused ^18^FDG PET/CT (**C**) image clearly demonstrates that the increased ^18^FDG uptake corresponds to the soft-tissue mass. Recurrent adenocarcinoma was histologically confirmed. Note unspecific ^18^FDG uptake in the morphologically normal right colon (physiologic variant).

Although considered potentially useful in the diagnosis and initial staging of colorectal cancer, currently there is no evidence of the superiority of ^18^FDG PET/CT over standard diagnostic work-up (Pelosi et al. 2007). However, ^18^FDG PET/CT has proven extremely useful in the assessment of patients with suspected recurrence. Early detection of recurrent disease is essential to perform an optimal salvage treatment and improve survival. Evaluation with CT of these patients is limited by its inability to differentiate between post-surgical or post-radiation scar tissue and recurrent disease, and to detect tumoral infiltration of normal-sized lymph nodes. This is particularly true for the evaluation of post-treatment presacral masses, which occur in an elevated proportion of patient and poses a clinical challenge (Fig. 8).

^18^FDG PET is very helpful in characterization of inconclusive lesions on morphologic imaging techniques, with reported diagnostic accuracies ranging from 74% to 95% as compared with 65% to 78% for CT (Schiepers et al. 1999; Huebner et al. 2000; Schaefer et al. 2007). ^18^FDG PET is also useful to localize occult metastatic disease in patients with rising tumor marker levels and negative conventional imaging tests, and identify unsuspected metastases in up to 25% of patients (Pelosi et al. 2007). There are limitations to PET, however. The lack of anatomic landmarks probably accounts for its relatively low specificity (76%) (Huebner et al. 2000), the false-positive interpretations of physiologic ^18^FDG uptakes in pelvis with post-treatment anatomic distortion (Even-Sapir et al. 2004), rendering it unsuitable for guiding biopsy, surgery of radiation. In addition, lesions below its spatial resolution (6mm) and those with low ^18^FDG uptake, such as mucinous adenocarcinomas can be missed on PET (Kamel et al. 2004).

Many of these drawbacks are overcome with ^18^FDG PET/CT. Several studies show the superiority of the combined modality in the detection of local recurrence of colorectal cancer, with sensitivities, specificities and diagnostic accuracies of 96%–100%, 96%–97% and 93%–96% respectively (Even-Sapir et al. 2004; Selzner et al. 2004; Schöder H et al. 2004; Votrubova et al. 2006; Pelosi et al. 2007). In a prospective study by Selzner et al. (Selzner et al. 2004) evaluating contrast-enhanced CT and unenhanced ^18^FDG PET/CT in metastatic colorectal cancer, both modalities yielded comparable sensitivities for the detection of liver metastases (95% and 91% respectively), but ^18^FDG PET/CT was superior for the diagnosis of intrahepatic recurrence after hepatectomy (50% versus 100%, p = 0.04), and extrahepatic disease (sentivities 64% and 89%, p = 0.02). Overall, ^18^FDG PET/CT has been reported to change therapeutic approach in up to 26% of patients with recurrent colorectal cancer (Selzner et al. 2004; Votrubova et al. 2006).

Despite the high accuracy, false-positive and false-negative findings may be encountered at ^18^FDG PET/CT. Post-surgical and post-radiation inflammatory tissue take up ^18^FDG. For this reason, ^18^FDG PET/CT imaging should be delayed until 2 to 3 months after completion of these treatments. Conversely, ^18^FDG PET/CT performed within one month of chemotherapy may yield false-negative results because neoplastic tissue might not be metabolically active. ^18^FDG PET/CT may not detect small lesions (< 5 mm) (Von Schulthess et al. 2006).

In addition, ^18^FDG PET/CT, particularly when performed only with unenhanced LD-CT, may nor suffice when resection of liver metastases is being considered. In these patients, further imaging with hepatic MRI of dual-phase contrast-enhanced multidetector-CT is usually required in order to improve lesion detection and provide adequate anatomic information.

To summarize, ^18^FDG PET/CT is extremely useful in the evalution of patients with suspected relapse of colorectal cancer, especially in cases with elevated carcinoembryonic antigen (CEA) and negative, equivocal or non-specific findings on conventional imaging techniques, and to characterize post-therapy presacral masses. Currently, ^18^FDG PET/CT is not indicated for screening or primary diagnosis or in patients with known diseminated disease. Promising uses of ^18^FDG PET/CT are monitoring chemo-radiotherapy and planning target volume in radiotherapic treatment (Ciernik et al. 2005; Pelosi et al. 2007).

### Lymphomas

Hodgkin’s lymphoma (HL) and non-Hodgkin’s lymphoma (NHL) are lymphoid neoplasias that have an elevated overall cure rate with current treatment modalities. Accurate staging is crucial for and adequate selection of therapy, and imaging plays an important role in the assessment of these patients. Enhanced CT has been the main modality used for staging and follow-up, but it is inaccurate for detecting involvement of normal-sized lymph nodes, spleen and bone marrow, and to exclude disease in post-therapy residual masses or enlarged-nodes. ^18^FDG PET is now widely used for staging and restaging of HL and agressive NHL, and has superseded gallium-67 scintigraphy as the modality of choice for metabolic imaging of these patients. Despite its high sensitivity and specificity, ^18^FDG PET has limitations related to the absence of anatomic precise localization of lesions, the non-specific ^18^FDG physiological uptake in some organs or in benign lesions, and the variable ^18^FDG avidity of several histologic types.

Current data in the literature suggest the improved performance of ^18^FDG PET/CT for staging and restaging of lymphomas as compared with contrast-enhanced CT (Freudenberg et al. 2004; Schaefer et al. 2004) and ^18^FDG PET alone (Allen-Auerbach et al. 2004), yielding a sensitivity of 91%–94% and a specificity of 88%–100%. ^18^FDG PET/CT precisely locates tracer activity to a specific organ or node, reducing the false-positive findings on ^18^FDG PET standalone scans, and facilitates the identification of sites of extranodal disease (Freudenberg et al. 2004; Schaefer et al. 2004; Schöder et al. 2004). ^18^FDG PET/CT has been reported to change the stage in up to 10% and 32% of patients as compared with ^18^FDG PET and CT respectively, and to alter patient management in up to 25% (Allen-Auerbach et al. 2004; Freudenberg et al. 2004; Raanani et al. 2006; Hernández-Maraver et al. 2006; Miller et al. 2006).

**Figure 9:**
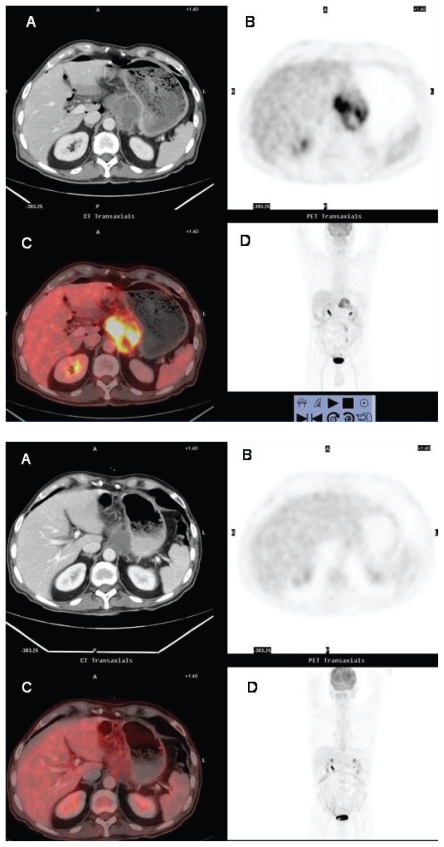
Assessment fo residual mass at completion of initial treatment in lymphoma. **9A:** Pretreatment ^18^FDG PET/CT study of a 27 year-old man with DLCB NHL. There is an abdominal mass that exhibits intense ^18^FDG uptake, consitent with lymphoma **9B:** Postreatment ^18^FDG PET/CT study shows persistent, though decresed in size abdominal soft-tissue mass, and complete resolution of the abnormal metabolic activity. The patient has remained free of relapse.

Probably, the most powerful application of ^18^FDG PET/CT in lymphomas is the post-therapy assessment, especially in patients with residual masses, enabling characterization as either fibrosis or viable lymphoma (Fig. 9). It allows earlier detection of residual or recurrent disease. In order to avoid false-positives caused by post-therapy inflammation, it is recommended to perform ^18^FDG PET/CT at least 3 weeks after chemotherapy, and 8–12 weeks after completion of radiation therapy (Juweid et al. 2007).

There is also evidence that mid-treatment ^18^FDG PET and ^18^FDG PET/CT are useful as prognostic indicators for disease-free and overall survival in HL and aggressive NHL, as early as after 1–3 cycles of chemotherapy (Jerusalem et al. 2000; Kostakoglu et al. 2002; Schot et al. 2003; Schaefer et al. 2007). Early identification of the patients who will not be cured with primary chemotherapy allows a change from a potentially toxic unsuccessful therapy to a more effective one. However, because of the false-positives that may result from post-chemoradiotherapy, and the morbidity and mortality associated with salvage treatments (including stem cell transplant), biopsy of the ^18^FDG-avid lesions is still needed (Schaefer et al. 2007). Other significant pitfalls regarding false-positive FDG uptakes after treatment include: opportunistic infections, thymic hyperplasia following chemotherapy, and bone marrow hyperplasia caused by colony-stimulating factor administration.

Thus, ^18^FDG PET/CT seems to be valuable to taylor therapy by separating subgroups of patients with worse prognosis who will benefit from different schemes of treatment, such as additional radiotherapy to areas of bulky disease or myeloablative chemotherapy followed by stem cell transplantation. Occasionally, ^18^FDG PET/CT may identify localized recurrence or residual disease that could be treated with radiation therapy.

False-negative findings have been described with lesions less than 1 cm, especially in the bases of lung, with small lesions in the liver or brain and mucosa-associated lymphoid tissue (MALT) NHL, because of the high ^18^FDG uptake in surrounding tissues, and also with low-grade lymphomas showing a low ^18^FDG uptake. In this regard, it is important to be aware of the histologic type of lymphoma when interpreting a ^18^FDG PET/CT. Although there exists considerable overlap in the intensity of ^18^FDG uptake, indolent lymphomas usually show lower ^18^FDG avidity than aggressive ones (Elstrom et al. 2003; Schöder et al. 2005). HL, difuse large B cell (DLBCL) NHL, and follicular lymphoma are consistently ^18^FDG avid. Conversely, peripheral T-cell, MALT, and small lymphocytic lymphoma exhibit variable, generally low ^18^FDG avidity, and may not be detectable on ^18^FDG PET scans. In this setting, the enhanced CT component of ^18^FDG PET/CT adds valuable information, and facilitates detection of low-intensity ^18^FDG uptake within lymph nodes or other lesions (Allen-Auerbach et al. 2004). On the other hand, ^18^FDG PET/CT may be useful in patients with low-grade NHL as an indicator of histologic transformation into a more aggressive disease (Schöder et al. 2005). If an unexpected increase in the intensity of ^18^FDG is detected in sites of disease with previously documented low uptake, ^18^FDG PET/CT may direct biopsy to such lesions in order to confirm transformation into a high-grade NHL.

Finally, the optimal protocol of ^18^FDG PET/CT for lymphomas is not yet determined. There is controversy as to the necessity of performing the CT component with intravenous iodinated contrast material (Schaefer et al. 2004; Raanani et al. 2006). Our own initial results (Rodríguez-Vigil et al. 2006) show a good correlation between unenhanced low-dose ^18^FDG PET/CT (LD-^18^FDG PET/CT) and contrast-enhanced full-dose ^18^FDG PET/CT (FD-^18^FDG PET/CT) for lymph node and extranodal disease, suggesting that LD-^18^FDG PET/CT might suffice as the only imaging modality in most patients with lymphoma, reducing contrast toxicity and radiation exposure. FD-^18^FDG PET/CT could be reserved for selected cases such as those with liver or splenic involvement. One approach could be to perform FD-^18^FDG PET/CT at initial staging and, unless the study shows ^18^FDG PET-negative lymphoma or hepatic or splenic involvement, continue performing LD-^18^FDG PET/CT on follow-up (Rodríguez-Vigil et al. 2006; Juweid et al. 2007).

### Malignant melanoma

Malignant melanoma has the potential to metastatize anywhere in the body, including unusual sites such as myocardium, meninges, and gastrointestinal tract, and shows one of the highest ^18^FDG uptakes of all tumors. For these reasons, whole-body^18^FDG PET has been proven to be highly effective for staging patients with high-risk melanomas (Eigtved et al. 2000; Tyler et al. 2000). However, metastases with no or weak ^18^FDG PET uptake may occur (Aquino et al. 2006). In addition the value of ^18^FDG PET imaging is limited for the depiction of metastases in the brain due to the high background activity of surrounding tissue, of small metastases, particularly in the lung, and of the necrotic lymph node metastases. The integrated ^18^FDG PET/CT modality avoids some of these false-negative findings at ^18^FDG PET. The diagnostic accuracy of PET/CT has been shown to be significantly higher than that of PET alone and CT alone for M-staging (0.98 vs 0.93 and 0.84 respectively), and significantly higher than that of CT for N-staging (0.98 vs 0.86), leading to a change in treatment in 48.4% of patients (Reinhardt et al. 2006). In a recent prospective study, Strobert et al. (Strobert et al. 2007) found that the added CT information improved the overall accuracy of integrated ^18^FDG PET/CT for depiction of melanoma metastases as compared with the readout on the basis of ^18^FDG PET information alone (96% vs 91%, p = 0.016). In 13% of patients metastases were detected only by using coregistered CT, especially metastases in the lung with no ^18^FDG accumulation.

^18^FDG PET/CT has become the standard diagnostic tool for patients with high-risk melanoma (Breslow thickness >1.5 mm or know metastases). On the other hand, ^18^FDG PET is less useful in patients without nodal or distant metastases (stage I–II) because of the higher sensitivity of sentinel node biopsy for detection of microscopic nodal metastases (Havenga et al. 2003).

### Head and neck tumors

Conventional imaging of head and neck tumors with CT and MRI that rely on morphologic changes is limited by their insensitive to detect metastases in normal-sized lymph nodes and early recurrences due to the post-therapy anatomic distortion and persistent contrast enhancement of benign tissue. Serial imaging is often needed to confirm stability of the lesion, suggesting scar or to evidence interval growth, indicating residual or recurrent disease, with a considerable delay in diagnosis and treatment. ^18^FDG PET has a higher sensitivity than MRI/CT, but the poor spatial resolution in this complex anatomy of the head and neck, and the variable physiologic uptake of ^18^FDG in normal structures such as muscles, brown fat, salivary glands, and the lymphoid tissue in the Waldeyer ring reduce its specificity and effectiveness (Blodgett et al. 2005; Fukui et al. 2005).

Enhanced ^18^FDG PET/CT has been reported to be superior to ^18^FDG PET or CT alone for the evaluation of malignancy in the head and neck, with overall sensitivity, specificity, and accuracy of 98%, 92% and 94%, against 74%, 75% and 74% of CT, and 87%, 91% and 90% of ^18^FDG PET respectively. ^18^FDG PET/CT showed an excellent negative predictive value (99%) (Branstetter et al. 2005). In a recent report, ^18^FDG PET/CT led to a TNM staging alteration in 34%, a change in radio-therapy planning technique and/or dose in 29%, and altered therapy response assessment in 43% of patients with squamous cell carcinoma of head and neck (Connell et al. 2007).

**Figure 10:**
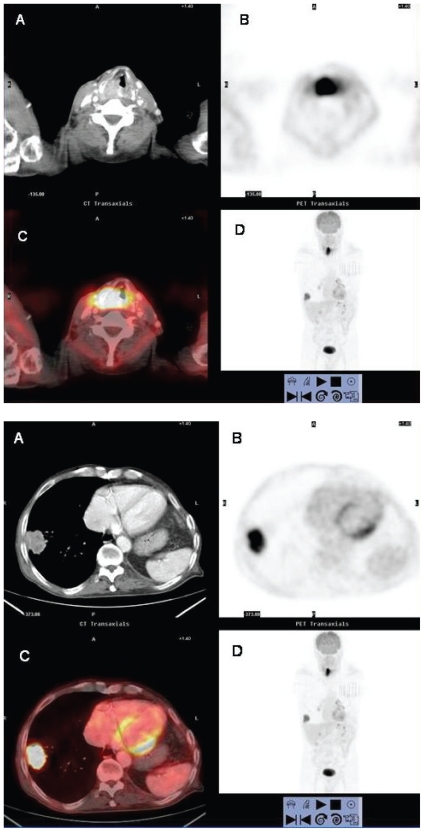
Synchronous lung cancer in a patient undergoing initial staging for laryngeal squamous carcinoma. **10A:** axial contrast-enhanced CT (**A**), axial PET (**B**) and fused axial ^18^FDG PET/CT (**C**) images at the level of vocal cords show a mass with intense ^18^FDG uptake in the larynx consistent with laryngeal carcinoma. A focus of increased tracer activity is also identified in the right lung at the coronal PET scan (**D**). **10B:** axial contrast-enhanced CT (**A**), PET (**B**), and fused ^18^FDG PET/CT (**C**) images at the lung bases. A peripheral lung mass abutting the pleura with a rim of increased ^18^FDG uptake is observed in the right lower lobe.

Indications of ^18^FDG PET/CT for head and neck cancer include: identification of unknown primary, initial staging for the tumor, nodes, and metastases, and detection of residual or recurrent disease after therapy (Funki et al. 2005; Hojgaard et al. 2007). It is also emerging as the method of choice for radiation therapy planning (Hojgaard et al. 2007). ^18^FDG PET/CT may be helpful in the search for a potential primary head and neck tumor in patients presenting with cervical metastatic adenopathies, and may direct biopsy in a second endoscopy. It has been reported that ^18^FDG PET/CT suggests the primary site in up to 68% of patients with unknown primary tumors (Gutzeit et al. 2005; Nanni et al. 2005; Wartski et al. 2007). ^18^FDG PET/CT allows a better depiction of the extent of the primary tumor, providing valuable information for the surgeon and/or radiotherapist. For regional nodal staging the main advantages of integrated ^18^FDG PET/CT are the ability to detect metastatic infiltration in normal-sized lymph nodes and better lymphadenopathy localization. In a recent study ^18^FDG PET/CT was superior to ^18^FDG PET and CT alone for predicting metastatic nodes on a level-by-level analysis (sensitivity 91.8%, specificity 98.9%, and accuracy 97.1%), and also for the pathological nodal classification (accuracy 85.1%) (Jeong et al. 2007). An additional advantage of performing whole-body ^18^FDG PET/CT is that it allows screening for distant metastases and synchronous second primary cancer. Although distant metastases are uncommon in head and neck cancers it is important to detect them because a large number of patients will receive loco-regional treatment only (surgery and/or radiation therapy). Second primary tumors are relatively frequent in this population, particularly lung and esophageal carcinomas because of the common risk factors for these neoplasias (Fukui et al. 2005; von Schulthess et al. 2005) (Fig. 10). In patients with early and advanced stage primary head and neck squamous cell carcinoma ^18^FDG PET/CT findings led to a change in the treatment plan in 31% of patients, mostly by upstaging (Ha et al. 2006). Probably, the most important application of ^18^FDG PET/CT in head and neck cancer is the assessment after treatment. Current data show a better performance of ^18^FDG PET/CT than either ^18^FDG PET or CT for detection of recurrent disease with sensitivities around 95%, and specificities of 60% (Zimmer et al. 2005; Fakhry et al. 2007). ^18^FDG PET/CT enables earlier detection of recurrences and with greater radiologists confidence than with CT alone (Fukui et al. 2005).

There are some limitations of ^18^FDG PET/CT in evaluating head and neck cancers. It is important to take into account the interval of time between ^18^FDG PET/CT and chemo-radiotherapy, as both, false-positive and false-negative findings have been reported when performed within 3 months of treatment. The optimal timing for post-therapy reevaluation is debatable, but it has been suggested that ^18^FDG PET/CT be performed at least 8–12 weeks after initial surgery or chemo-radiotherapy in order to obtain a more reliable malignancy status evaluation (Hojgaard et al. 2007). The sensitivity of ^18^FDG PET/CT decreases for tumors smaller than 1 cm, especially flat mucosal lesions or those near normal structures displaying physiologically high ^18^FDG accumulation (brain, tonsils). In addition, the ^18^FDG uptake may be underestimated in small lesions owing to partial volume averaging with normal tissue and show apparentely benign values. On the other hand, some slow-growing salivary gland tumors (mucoepidermoid, adenoid cystic tumors), and spindle cell neoplasms may have a low avidity for ^18^FDG and yield false-negative results. Also lymph nodes with extensive necrosis may show low tracer uptake and cause false-negative findings (Fukui et al. 2005).

## Other Indications and Other Tracers

^18^FDG PET/CT is also indicated in the staging and restaging of iodine-negative thyroid cancer: dedifferentiated papillary or follicular cancer (neoplastic cells lose their ability to accumulate iodine), and advanced Hürtle cell and medullary carcinoma (Schmid et al. 2003).

^18^FDG PET/CT is also a useful imaging modality in the assessment of response to treatment and detection of relapse in other metabolically active cancers, such as musculoskeletal sarcomas, gastrointestinal stromal tumors and esophageal carcinomas. ^18^FDG PET/CT has proved useful in the detection of metastases that can be unsual in appearance or in unexpected locations in patients with esophageal carcinoma (Bruzzi et al. 2007). It has been reported to be superior to PET or CT alone and change patient management in up to 22% by detecting nodal and organ metastases (Schöder et al. 2004; Weber et al. 2004).

Though well-differentiated hepatocellular carcinomas (HCC) do not consistently show increased ^18^FDG uptake, ^18^FDG PET/CT may be useful in patients with poorly differentiated HCC, particularly in depiction of distant metastases of post-therapy recurrence (Von Schulthess et al. 2006). Furthermore, it may have a role in the selection of liver transplantation candidates. Yang et al. (Yang et al. 2006) have found that ^18^FDG PET was a good preoperative tool for predicting post-transplantation tumor recurrence in these patients.

Preliminary studies also suggest that ^18^FDG PET/CT may be valuable for ovarian, cervical, and endometrial cancer (von Schulthess et al. 2006).

Other PET tracers more tumor-specific than ^18^FDG are being explored for certain cancers. These new tracers depict amino acid metabolism, receptor density, tissue hypoxia, angiogenesis and apoptosis, and could prove valuable in tumors with low avidity for ^18^FDG, such as prostate cancer. ^18^F-choline and ^18^F-ethyl coline may become useful in prostate carcinoma staging (von Schulthess et al. 2006). Amino acids labeled with radionuclides such as ^11^C-methionine, ^18^F-ethyl tyrosine, ^18^F-fluoro-alpha-methyl tyrosine (FAMT) and ^18^F-thymidine are markers of protein synthesis (Oriuchi et al. 2006). They are potentially more cancer-specific than ^18^FDG as they do not accumulate in inflammatory tissue, but are less sensitive for tumor staging. FAMT has shown promising results in detection of pancreas, liver and brain tumors (Inoue et al. 2001) because it does not show intense accumulation in these organs. Also, octreotide derivates and ^18^F-DOPA are being investigated as markers of neuroendocrine tumors (von Schulthess et al. 2006).

To summarize, ^18^FDG PET/CT has emerged as powerful imaging tool in clinical oncology due to the synergistic advantages of its components. It has become the new standard imaging modality for many types of cancer. Despite its benefits, ^18^FDG PET/CT has recognized limitations, and some clinical questions remain open. Ongoing research programmes will probably cast light on these issues. In addition, the application of new PET tracers other than ^18^FDG that target specific biological characteristic of various cancer cells holds promise for further improvements in the management of cancer patients.
